# Supplementary material for: 200 GHz single chip microsystems for dynamic nuclear polarization enhanced NMR spectroscopy
Source: Nat Commun. 2024 Jun 28;15:5485. doi: 10.1038/s41467-024-49767-z (PMC11213862; doi:10.1038/s41467-024-49767-z)
Supplement: Supplementary file 1 — Supplementary Information [file 41467_2024_49767_MOESM1_ESM.pdf]

Supplementary Information
200 GHz single chip microsystems for dynamic nuclear polarization enhanced NMR spectroscopy

Nergiz Sahin Solmaz*, Reza Farsi, Giovanni Boero

Institute of Electrical and Micro Engineering (IEM) and Center for Quantum Science and Engineering (QSE)

École Polytechnique Fédérale de Lausanne (EPFL), 1015 Lausanne, Switzerland

Supplementary note 1: Isolation between NMR and ESR microcoils

The electromagnetic coupling between the concentric ESR and NMR integrated microcoils is computed using an EM solver (ADS Keysight, Momentum Microwave). The S-parameters file generated by this simulation is imported to Cadence using the n-port cell of the analogLib. To emulate the ESR excitation condition, a simulation is performed as in Supplementary Fig. 1. With a 200 GHz current I_{IN} of 28 mA, which is the current delivered by the microwave oscillator into the ESR microcoil, the induced voltage at the input of the LNA of NMR receiver ($V_{IN,P} - V_{IN,N}$) is 1.5 mV and the voltage at the output of the LNA ($V_{OUT,P} - V_{OUT,N}$) is 0.15 mV. This simulation shows that the effective isolation between the ESR and NMR microcoils is sufficiently large to avoid the saturation of the NMR receiver by the continuous wave ESR excitation. This large effective isolation can be attributed to: 1) the self resonance frequency of the NMR microcoil which is around 1 GHz, i.e. much smaller than the ESR excitation frequency of 200 GHz, 2) the ESR microcoil has a diameter of about 45 μm, i.e. significantly smaller than the 200 μm diameter of the NMR microcoil.

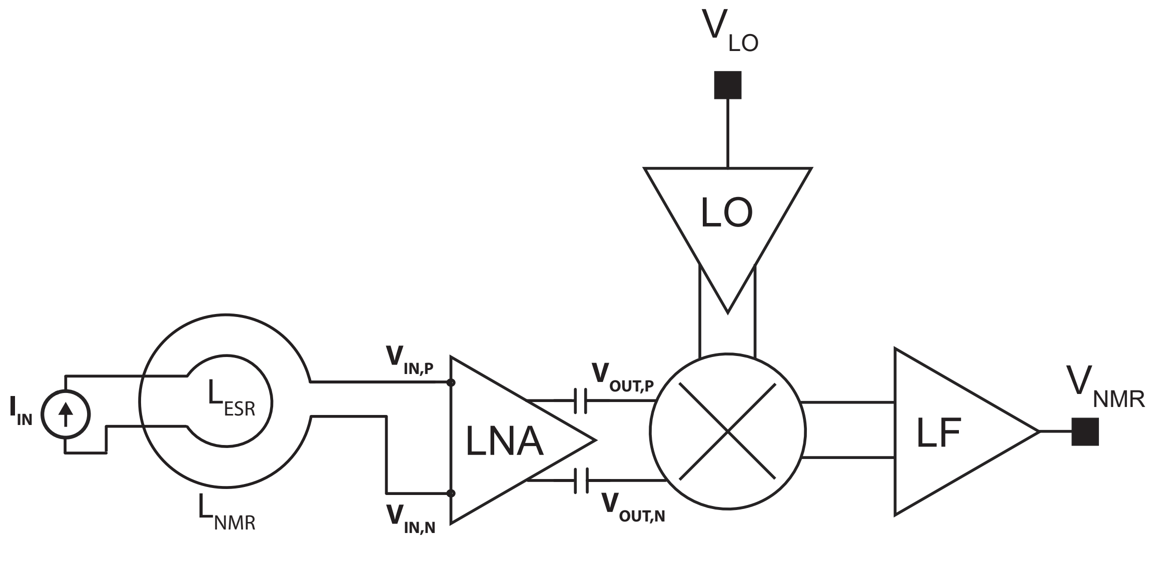

Supplementary Figure 1: Bloch diagram of the NMR receiver used in isolation simulation. I_{IN} represents the microwave current produced by integrated oscilllator that runs in the ESR microcoil during the ESR excitation.

*Corresponding author
Email address: nergiz.sahin@epfl.ch (Nergiz Sahin Solmaz)

Supplementary note 2: LNA gain and noise

The LNA gain and noise are simulated using Cadence Spectre. The concentric ESR and NMR microcoils are simulated using an EM solver (ADS Keysight, Momentum Microwave). As mentioned in the main text, at 300 MHz, the NMR coil inductance is about 79 nH and it series resistance is about 24 Ω. The S-parameters file extracted from the EM simulation is imported to the Cadence environment to include the effect of the microcoils on the gain and noise simulation. Supplementary Fig. 2 shows the configuration which is used in this simulation. The gain of the LNA is calculated as $(V_{OUT,P} - V_{OUT,N})/V_{IN}$, which represent the effective gain for the electromotive force induced in the NMR microcoil by the spin precession (i.e., the NMR signal). The input referred noise is computed dividing the $(V_{OUT,P} - V_{OUT,N})$ noise spectral density by the gain. The presence of the ESR microcoil has a neglible effect on the gain and noise. The gain and the input refered noise of the LNA are shown in Supplementary Fig. 3.

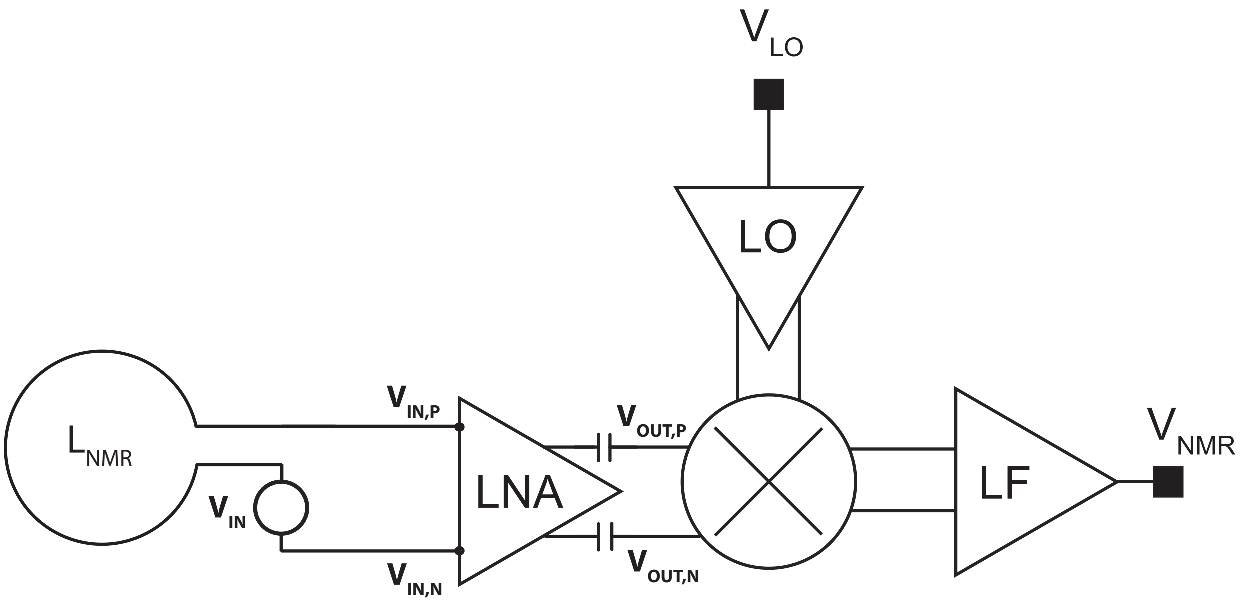

Supplementary Figure 2: Bloch diagram of the NMR receiver. The NMR microcoil L_{NMR} is connected to the LNA directly, i.e., without tuning/matching capacitors. V_{IN} represents the electromotive force induced in the NMR microcoil by the spin precession (i.e., the NMR signal). The transistor level schematics of the LNA, mixer, LO amplifier, and LF amplifier are given in Supplementary Fig. 5.

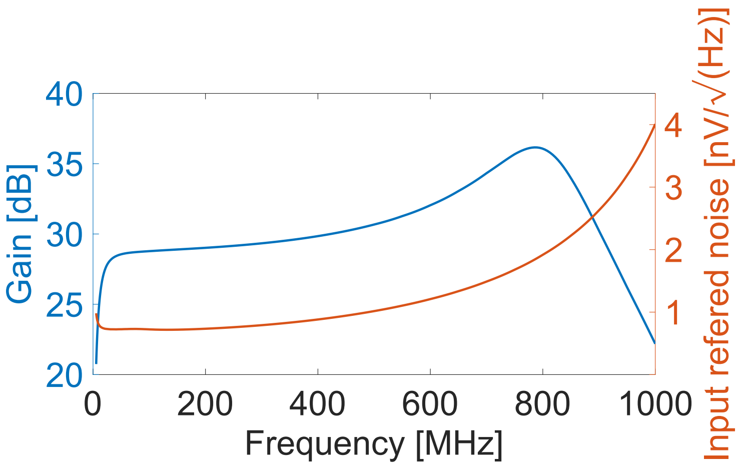

Supplementary Figure 3: Simulated gain and noise of the LNA. The input and output voltages used in the computation of the gain and noise are defined in Supplementary Fig. 2. The gain of the LNA is calculated as $(V_{OUT,P} - V_{OUT,N})/V_{IN}$, which represent the effective RF gain for the electromotive force induced in the NMR microcoil by the spin precession (i.e., the NMR signal). The input referred noise is computed dividing the $(V_{OUT,P} - V_{OUT,N})$ noise spectral density by the gain as defined above.

Supplementary note 3: Pulse sequence

All NMR and DNP enhanced NMR experiments reported in this work are conventional free induction decay (FID) measurements performed after a single $\pi/2$ pulse. The microwave excitation from the integrated ESR oscillator is permanently on during all measurements. The pulse sequence diagram is given in Supplementary Fig. 4. The microwave frequency is set by the tuning voltage V_T, supply voltage VDD_{ESR}, and the bias current I_B of the single chip integrated microwave oscillator.

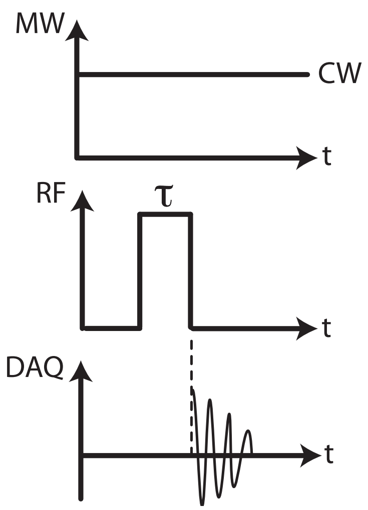

Supplementary Figure 4: Pulse sequence diagram used for all NMR and DNP enhanced NMR experiments. MW: microwave excitation, CW: continuous wave, RF: radio frequency excitation, DAQ: data acquisition. The microwave excitation is a CW excitation at about 200 GHz. The RF excitation is a pulsed excitation at about 300 MHz, with a pulse lenght τ of about 10 μs. The specific values for the MW frequency, the RF frequency, and pulse lenght for each performed experiment are given in the main text.

Supplementary note 4: Detailed description of the NMR receiver

The block diagram of the NMR receiver and the detailed transistor level schematics of the LNA, mixer, low frequency (LF) amplifier, LO amplifier, and their building blocks are shown in Supplementary Fig. 2 and Supplementary Fig. 5, respectively. The NMR microcoil L_{NMR} is connected to the LNA directly (i.e., without tuning/matching capacitors). A differential topology is chosen for the LNA and for the complete receiver architecture to have a higher immunity against noise and interferences. The gain of the LNA is limited to 28 dB to reduce the risk of oscillations. A cascode topology is chosen to achieve higher gain and reverse isolation without significantly impacting the power consumption and the noise [1]. The LNA operates from DC to 1 GHz and has a total input referred noise of 0.8 nV/Hz$^{1/2}$ at 300 MHz, including the thermal noise of the 24 Ω series resistance of the NMR microcoil (see Supplementary Fig. 3). The LNA is connected to the down-conversion mixer with decoupling capacitors which sets the minimum operating frequency of the receiver chain to 15 MHz. A double-balanced mixer with Gilbert cell topology is used to downconvert the NMR signal frequency. The local oscillator (LO) signal for the mixer is brought to the chip from outside of the magnet. On the chip, the LO signal is split and amplified by the LO Amplifier, producing two signals having 180° phase shift as seen in Supplementary Fig. 5. The R_{11} and R_{12} resistors are used to bias the DC operating points of the inputs of the first stage of the LO Amplifier. After the first stages, the LO_N output is obtained by using four inverters in series whereas the LO_P output is obtained by using three inverters and a delay cell in series. The output of the mixer is directly connected to the LF amplifier. A resistive feedback is used to set the gain of the LF amplifier. The LF amplifier consists of an operational amplifier (OPAMP) that is designed in the Miller operational transconductance amplifier (OTA) topology. The design of the current source in the OPAMP is based on the one described by Yoo et al [2]. Frequency down-conversion enables the use of an LF amplifier on the same chip to increase the overall gain of the NMR receiver to 75 dB. The LF amplifier has a bandwidth of 4 MHz and an output impedance of 6 Ω. The large LF bandwidth is important for experiments on solid samples having short T_2^* which requires a receiver chain with short deadtimes after the excitation pulse.The measured deadtime is about 1 µs. The simulated overall gain and noise of the NMR receiver is given in Supplementary Fig. 6.

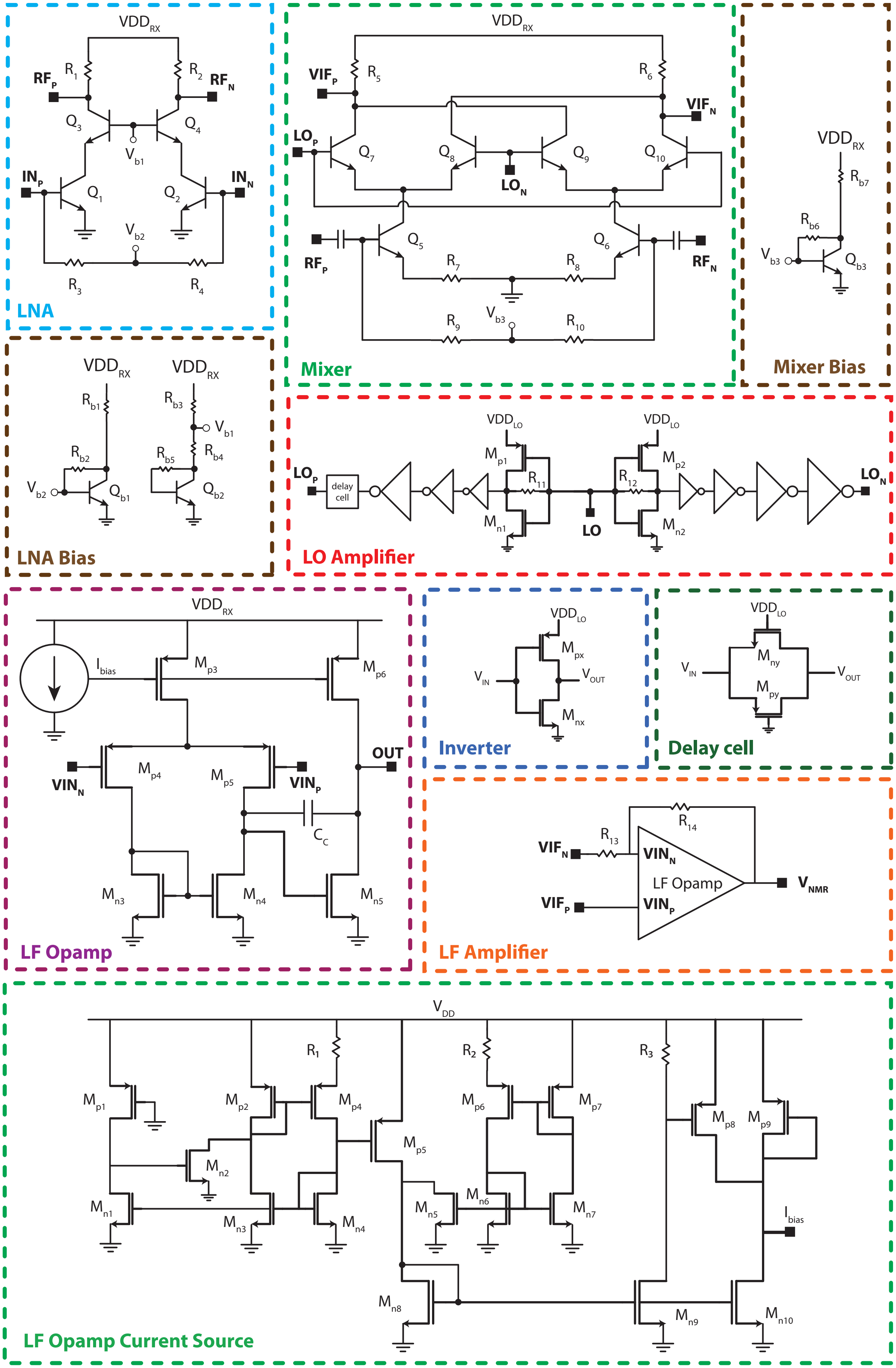

Supplementary Figure 5: Detailed schematics of the NMR receiver.

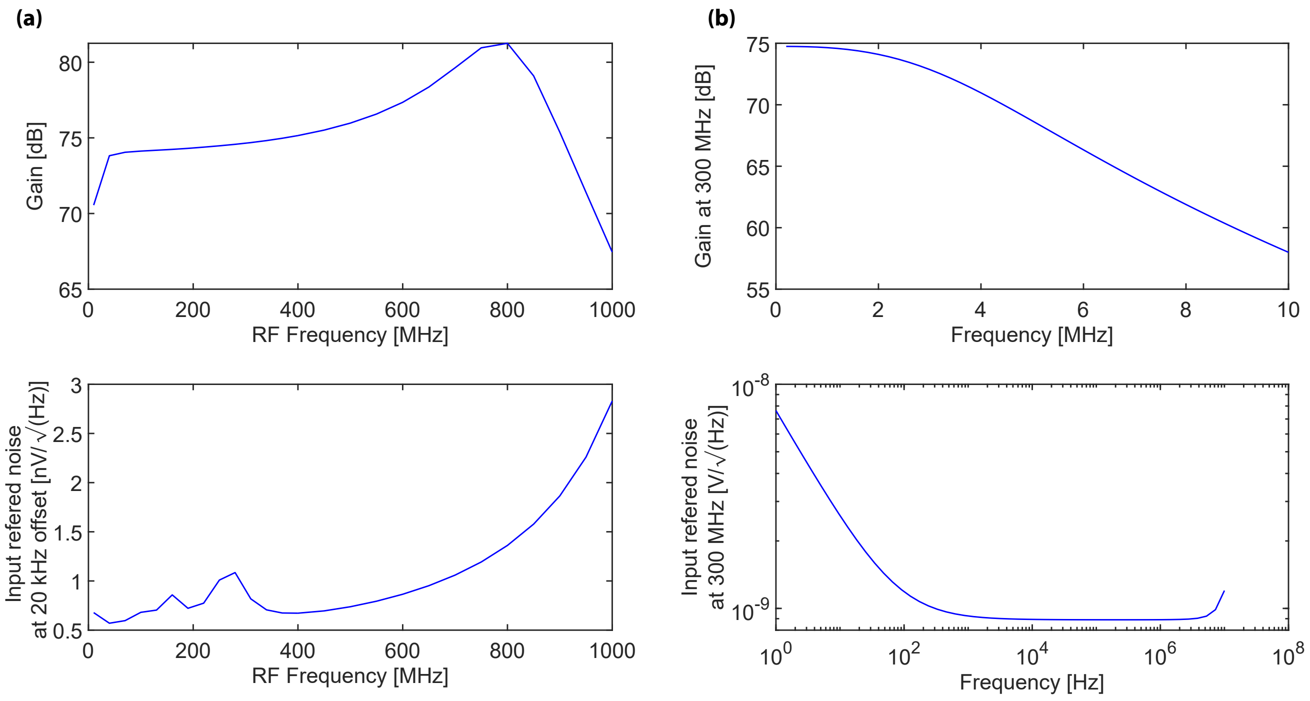

Supplementary Figure 6: The simulated gain and input referred noise of the NMR receiver (a) vs RF frequency (b) vs LF frequency.

Supplementary note 5: Microwave currents of the oscillator array

The oscillator array consists of four oscillators which are electromagnetically coupled as shown in Supplementary Fig. 7(a) and (b). The coupling is simulated using an EM solver (ADS Keysight, Momentum Microwave). The S-parameters file extracted from the ADS simulations are imported to Cadence to compute the currents in the four oscillators. These simulations show that all the oscillators are frequency and phase-locked, and the microwave currents running in the ESR microcoils are shown with arrows in Supplementary Fig. 7(b). Hence, the array of ESR microcoils produces a field which is similar to the one produced by a microcoil having a diameter two times larger than the single microcoil (shown in red lines in Supplementary Fig. 7(b)), except in the central region between the microcoils. The microwave magnetic field generated by the array of microcoils is approximately two times smaller than the one generated by the single microcoil.

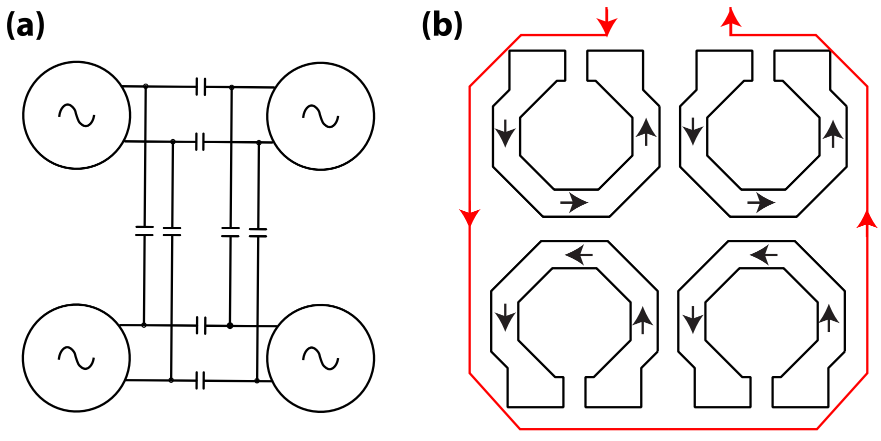

Supplementary Figure 7: (a) Capacitive coupling of the four ESR oscillators in the oscillator array microsystem, (b) Microwave current directions in the array of microcoils.

Supplementary note 6: Details on the design and production of DNP microsystems

The two integrated circuits are realized using a 130 nm SiGe technology (IHP SG13G2Cu). This technology is accessible throughout Europractice (https://europractice-ic.com/technologies/asics/ihp/). The price for the fabrication of 40 identical chips each having an area of about 1 mm^2 is about 5000 Euros. This is the price for a production of 40 chips. The mass production of such chips would reduce the cost down to the 1 Euro/chip level. The time needed for the design of the chips reported in this work was about three months for one full-time-equivalent Ph.D. student with previous experience in integrated circuit design. The time interval between the submission of the design to receiving of the fabricated chips was about six months.

References

[1] J. W. Rogers, C. Plett, Radio frequency integrated circuit design, Artech House, 2010.

[2] C. Yoo, J. Park, Cmos current reference with supply and temperature compensation, Electronics Letters 43 (25) (2007) 1422–1424.
